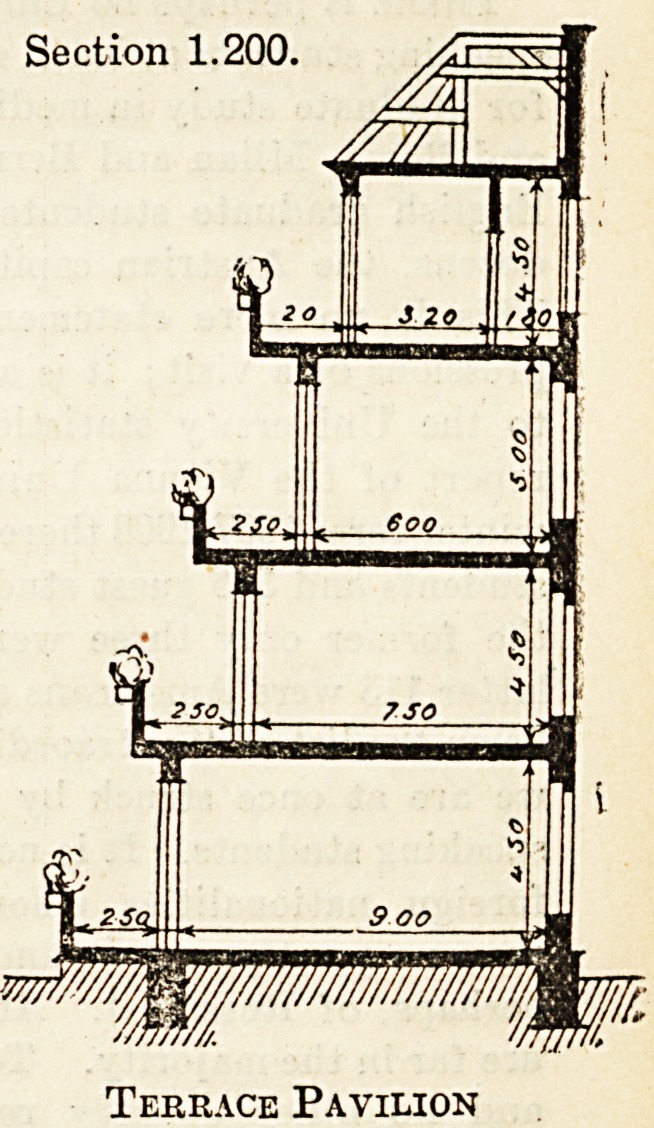# The Balcony Question in Hospitals

**Published:** 1908-10-31

**Authors:** 


### HOSPITAL ADMINISTRATION.

### CONSTRUCTION AND ECONOMICS.

### THE BALCONY QUESTION IN HOSPITALS.

A NEW SUGGESTION.

The adequate provision of balcony space in new hospitals is a matter that has hitherto been treated in a stepmotherly fashion by designers and builders. Usually it is deemed sufficient to construct a covered balcony, opening from the ward, and the variations of this system are numerous in the extreme, though none of them can be said to be ideal. In single-storied buildings of the pavilion type the difficulties are easily overcome, and as ideal examples the fine balconies, or rather terraces, with which each ward is provided at the Hamburg Eppendorf general hospital may be brought forward. In buildings of the corridor type, or of the "double-storied pavilion block system" which is now again coming into favour, especially in the new Austrian and Italian hospitals, the balcony remains what it was fifty years ago. To take, for instance, our London hospitals, none of them can be said to possess an ideal balcony. The small covered hedges, commanding the Houses of Parliament and the river, which St. Thomas's provides for its patients can scarcely be deemed ideal. Still less, to take another instance, can one be loud in admiration of the comparatively new balconies added to the right surgical wing of Guy's Hospital. These latter are damp, dark, and covered; they share with all balconies in the corridor buildings (except those on the top story, which are ideal in many ways, as the balcony attached to the Guy's "attic ward" overlooking the park) the disadvantage of receiving no overhead sunlight, and in many cases they are not directly connected with an emergency staircase. The difficulty hitherto has been mainly to overcome the first objection, and various methods have been proposed. The most feasible seems to be the plan adopted at some sanatoria, where the balconies are not built directly underneath each other, but at different parts, so that no one is directly overshadowed by its fellow above. This system has obvious disadvantages, not the least of which is the expense entailed by such construction and the amount of room required, which is usually taken at the expense of the ward itself.

At the fourteenth international Congress for Hygiene held at Berlin, Dr. Sarason read a paper in which he outlined a new system, intended to overcome these difficulties by building the hospital in what he proposes to call the "Terrace Style." The accompanying sectional designs, taken from his paper, "Ein neues Bausystem für Krankenanstalten und Wohnhaüser," in the *Gesundheits-Ingenieur*, explains the principles of this suggested system better than any lengthy description would be able to do:

Fig. 1 shows the sectional plan of a corridor building designed for a sanatorium, while Fig. 2 shows the corresponding design for a corridor hospital. It will be seen that the balcony space is almost entirely free, *i.e.* the amount of shadow from the overhead balcony on the balcony below is reduced to a minimum. This is obtained by pushing the ward back, room being made by diminishing, not the ward space itself, but the corridor space. The amount of room thus obtained is considerable, even when only a limit of three feet is available.

The suggested modification of Dr. Sarason is a practical attempt to deal with a difficulty which is met with not only in hospital construction but in the building of large flats and dwelling houses. One has only to inspect our London flats to find how ill-provided they are, for the most part, with balconies that can really be termed open-air terraces. Where these balconies overlap they are poor make-shifts, and the dwellers on the first and middle floors get practically no free overhead lighting. Those on the top story are, of course, much better off. They receive a full amount of air and sunlight, but they get these benefits

Terrace Sanatorium

Terrace Pavilion at the expense of their neighbours below. Dr. Sarason's paper, which space forbids us publishing in full, deals fairly with the advantages of the new system. The author points out, justly, that this question of sunlight is an important one in artisans' dwellings. The ideal pavilion system, where every building is one-storied, and stands in a domain of its own, strictly isolated from its fellows, is for the most part impracticable owing to its vast expense and the extravagance of ground space it entails. Where it has been adopted on a large scale (as in the Virchow Hospital of Berlin) its advantages are questionable, owing to the expense its administration entails. In city dwellings it is practically impossible to adopt the pavilion system—even at Letchworth it has not been attempted. On the other hand, the Terrace building is a practical compromise which bids fair to become a favourite system in the near future. Those interested in the new system will, of course, be anxious to find out what are its practical possibilities. So far as we know, no hospital or artisans' dwelling has as yet been constructed on the Sarason system. The author, however, has prepared plans and specifications for various buildings designed in accordance with his proposals. Thus in an exhaustive work, now in course of publication, he has dealt with no fewer than ten different types of buildings to which his suggestions are applicable. These range from corridor hospitals for 100 beds and large artisans' dwelling flats, to smaller private dwelling houses. The work is complete with details and specifications, and as the system has been patented by Dr. Sarason, those interested in hospital and dwelling-house construction will be able to find authentic first-hand information on the subject from the author himself.

The system appears to us to be capable of modification in some respects. Such modifications will suggest themselves to every builder in individual cases, and it is possible that with a little ingenuity the system of terrace balconies may also be made to serve for already existing institutions which are at present inadequately provided with open air and sunlit balconies. A building such as Dr. Sarason has modelled deserves cordial praise, for it certainly deals very efficiently with a problem which is daily becoming more difficult as our cities grow larger. To a slight extent our "Ancient Lights" protects us from the excessive encroachment on air and sun space, which is so marked a feature in buildings in the larger Continental cities; but even in our suburban dwellings the amount of sunlight that is available for the inmates is hardly adequate. There is always the back garden in the suburban villa, but, as Dr. Sarason points out, it is not generally made full use of. The balcony or verandah is, after all, the most attractive addition to the dwelling house; it certainly is one of the most hygienic additions that the architect can plan, always provided that it is not covered in and encroached upon to such an extent that it practically becomes an interior.

## Figures and Tables

**Figure f1:**
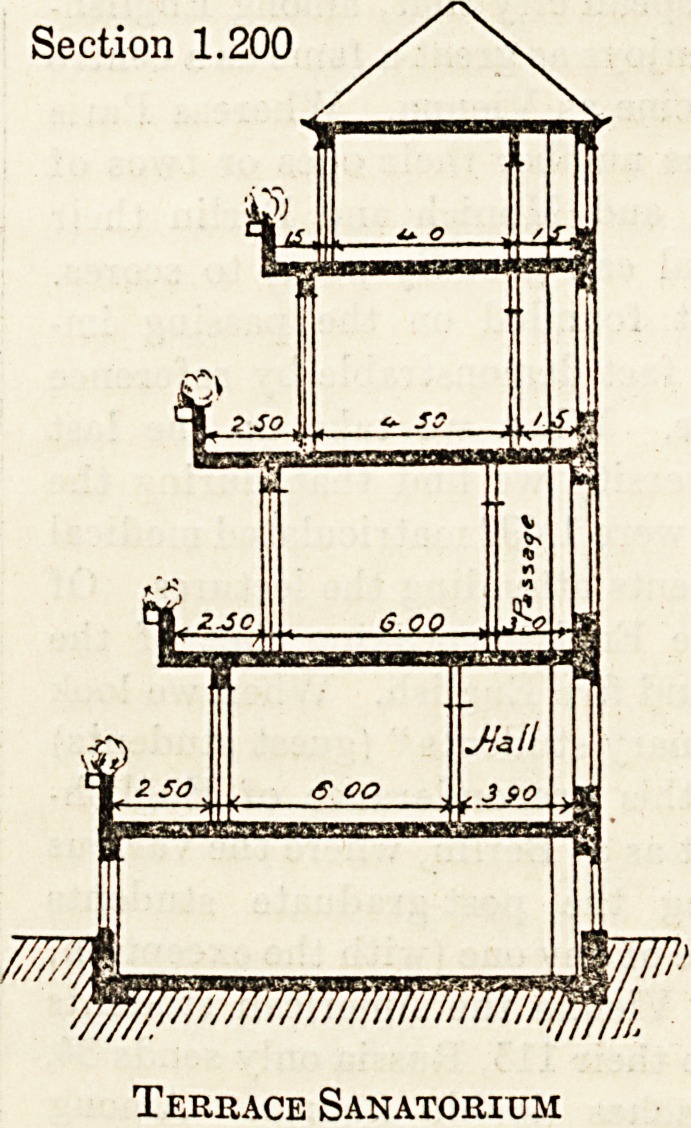


**Figure f2:**